# CMV-DNA detection in patients with thrombocytopenia

**DOI:** 10.1590/S1516-31802002000200008

**Published:** 2002-03-02

**Authors:** Valder Roberval Arruda, Gislaine Borba Oliveira, Joyce Maria Annichino-Bizzacchi, Paula Durante, Fernando Ferreira Costa, Sandra Cecília Botelho Costa

The production of platelet autoantibodies may have an idiopathic pathogenesis or may occur in conjunction with other autoimmune diseases, cancer, the use of certain drugs^1^ or infections such as human immunodeficiency virus (HIV) and cytomegalovirus (CMV), resulting in secondary autoimmune thrombocytopenia.^1,2^ Recently, we reported an unusual case of severe thrombocytopenia associated with CMV infection in a healthy person whose response to treatment with ganciclovir was followed by an improvement in the platelet count.^3^ Based on this finding, we have assessed the prevalence and possible clinical implications of CMV-DNA positive patients with thrombocytopenia attended at a university teaching hospital.

From June 1996 to January 1997 we studied 84 patients with chronic thrombocytopenia (63 females and 21 males). The median age of these individuals was 37.4 years (range: 3 to 82 years) and the median follow-up was 25 months (range: 2 to 140 months). Idiopathic thrombocytopenia purpura (ITP) was diagnosed in 61 out of the 84 patients (73%). In the remaining 23 patients, the cause of thrombocytopenia was a systemic autoimmune disease in 15 cases, drug consumption in four cases, related to HIV infection in two cases, and cancer in two cases. All patients were screened for the presence of CMV-DNA.

Genomic DNA was extracted from peripheral blood and the CMV-DNA was amplified by nested polymerase chain reaction (PCR) using the primers described by Demmler et al.^4^ Amplification of the b-globin gene was used as an internal control. CMV-DNA was detected in two out of the 84 patients. In both of these patients, there was a concomitant HIV infection.

Thrombocytopenia is a common hematological complication which affects almost half of HIV-infected patients.^5^ In one of the two CMV-positive patients with HIV, the platelet count returned to normal only after specific antiviral therapy with ganciclovir. The improvement in the platelet count only after treatment with ganciclovir suggests that the CMV infection was probably the cause of this thrombocytopenia.

The demonstration of CMV-DNA by nested-PCR is very useful in this situation because of the difficulty in detecting CMV-IgM antibody in immunosuppressed patients, particularly those with acquired immune deficiency syndrome (Aids).^6^ The absence of CMV-DNA in the other patients confirmed the rarity of thrombocytopenia occurrence caused by CMV infection among non-immunosuppressed individuals.^3,7^ The detection of CMV infection in patients with thrombocytopenia and an HIV infection should provide correct diagnosis of the primary cause of the platelet disorder and may allow the use of specific therapy. We suggest that screening for CMV-DNA among patients with thrombocytopenia associated with an HIV infection could be a useful addition to the general diagnostic guidelines already proposed.^5^
